# Cellulose Isolated from Giant Miscanthus by Nitric Acid Treatment with Acid Recycling

**DOI:** 10.3390/polym18192334

**Published:** 2026-09-24

**Authors:** Yulia A. Gismatulina, Vera V. Budaeva, Vladimir N. Zolotukhin, Gennady V. Sakovich

**Affiliations:** Bioconversion Laboratory, Institute for Problems of Chemical and Energetic Technologies, Siberian Branch of the Russian Academy of Sciences (IPCET SB RAS), Biysk 659322, Russia

**Keywords:** cellulose, plant cellulose, miscanthus stems, *Miscanthus* × *giganteus*, nitric acid treatment, recycling

## Abstract

Stems of giant miscanthus―a perennial grass crop―were investigated in this study. The Kürschner cellulose content was determined to be 52.8 wt%, indicating that this feedstock holds considerable promise for cellulose isolation. For the first time, cellulose was isolated from giant miscanthus stems via a modified nitric acid process involving treatment of the feedstock with nitric acid at the first stage with fivefold acid recycling. The isolated cellulose samples were characterized by consistently high and mutually comparable quality. A high α-cellulose content of 92.0–94.0 wt% and a high degree of polymerization of 1750–1850 were achieved, along with minor contents of non-cellulosics: 0.41–1.06 wt% acid-insoluble lignin, 3.12–3.82 wt% pentosans, and 0.13–0.35 wt% ash. This method ensures the isolation of high-quality cellulose in a 36% yield. At the same time, the content of non-cellulosics were reduced manifold, specifically: lignin by 24.6–63.7 times, pentosans by 4.0–6.2 times, and ash by 5.9–15.9 times, which corresponds to their extraction degree of 98%, 81% and 90% on average, respectively. A pooled cellulose sample was characterized by SEM, IR spectroscopy, and TGA/DTA/DTG. The obtained results justify the use of the modified nitric acid method with fivefold recycling of the spent nitric acid solution, which would reduce reagent and water consumption without compromising the quality attributes of the resulting cellulose. The practical value lies in the experimental demonstration that high-polymer cellulose can be obtained from giant miscanthus with a degree of polymerization reaching 1850, which significantly expands the potential applications of cellulose and its chemical modification products.

## 1. Introduction

The growing global demand for environmentally friendly and sustainable materials has intensified interest in optimizing cellulose extraction processes from various types of feedstock, including woody and non-woody plants, agricultural waste, logging residues, marine biomass, and fruit and food waste [1,2,3,4]. Among natural biopolymers, cellulose is the most abundant organic compound on Earth, accounting for 40–50% of the total biomass. Its annual biosynthesis exceeds 1.5 trillion tons, representing a vast renewable resource [4,5].

Cellulose, a complex linear polysaccharide, is the main structural component of plant cell walls and plays a key role in providing mechanical strength and rigidity to plant tissues [6,7,8,9]. Owing to its unique properties, cellulose finds wide application in the pulp and paper, textile, chemical, and pharmaceutical industries, as well as in renewable energy production, and its scope of application continues to expand year by year [1].

The choice of feedstock and the selection of the isolation method significantly affect the quality and yield of cellulose, since strong interactions with hemicelluloses and lignin make cellulose isolation from lignocellulosic biomass a challenging task [10,11,12].

The excessive utilization of conventional cellulose sources—cotton and wood—has led to the search for alternative feedstocks [13,14]. Due to the growing demand for environmentally friendly and sustainable materials derived from plant biomass, research aimed at improving the efficiency of isolation processes for multifunctional cellulose from various types of feedstock, including non-woody plants, agricultural waste, logging residues, fruit waste, aquatic plants, and marine algae [4,15,16,17,18,19,20,21,22,23,24,25,26,27], has shaped a fundamental research trend worldwide.

Research on the conversion of energy crops, particularly Miscanthus, into valuable and economically viable products is currently at the forefront [8,15]. Miscanthus is considered one of the most promising sources of cellulose, as it is capable of utilizing external resources more efficiently than C3 plants and many C4 plants [8].

In this work, giant miscanthus (*Miscanthus* × *giganteus*)—a perennial grass crop with a cellulose content of 35–50% and an annual biomass yield of up to 40 t/ha over a period of 18–25 years—was chosen as the substrate for study [8,16,28,29]. The choice of miscanthus is driven by its wide global spread, resistance to early frosts and pests, and its annual capacity to produce substantial biomass yields even when cultivated on marginal and degraded lands, which contributes to sustainable feedstock supply and resource use efficiency for high-performance applications in modern industries. Giant miscanthus attracts particular interest as a popular, industrially significant crop with enormous potential for chemical and biotechnological conversions [30].

The conventional methods for cellulose isolation often involve harsh chemical treatment, which cause environmental concerns, affect the quality of isolated cellulose, and require additional measures to meet environmental standards [12]. To address these issues, researchers worldwide are developing alternative, safer approaches for cellulose isolation, primarily from readily renewable plant feedstocks without using aggressive reagents [14,16,17,18,19,20,21,22,23,24,25,26,27,31,32,33,34].

Since lignin from non-woody feedstocks and, specifically, miscanthus, differs significantly from wood lignin [35], cellulose can be isolated from miscanthus using more environmentally benign (green) technologies, in particular, without sulfur and chlorine reagents, and consequently, with a minimal adverse environmental impact [36,37,38]. It was noted [39] that even when miscanthus biomass is combusted, the amount of harmful substances released does not exceed those absorbed during growth, which enables a closed carbon cycle.

In the present study, the nitric acid method was chosen for cellulose isolation, which involves sequential treatment of the feedstock with dilute nitric acid and sodium hydroxide solutions. This method has proven effective for obtaining high-quality cellulose from another miscanthus species [40]. It should be noted that the other researchers have also employed a dilute nitric acid solution at the first stage of cellulose isolation from plant feedstocks.

Owing to the low concentrations of the solutions (not exceeding 4%), the use of aggressive reagents, such as nitric acid and sodium hydroxide, makes this cellulose isolation method quite technological and resource-saving, since the process is carried out in simple, accessible, sealed equipment at atmospheric pressure and a temperature below 100 °C. The equipment is fitted with a reflux condenser to trap and return nitrogen oxides back into the cooking liquor. Nitrogen oxides are released during the main stage of the process—the nitric acid treatment of the feedstock. Furthermore, after neutralization with ammonia or potassium hydroxide, the spent cooking liquor constitutes a solution of a lignin–humic fertilizer complex, including fulvic acids [41,42].

The most important feature of this method is its economic feasibility in terms of reagent and energy costs, in contrast to feedstock pretreatment at temperatures above 100 °C. This feature is decisive in the development of a cost-effective process. Recycling—the repeated use of the spent cooking liquor for subsequent treatments of fresh feedstock portions—can be applied in order to further improve the economic efficiency of the process. The recycling of liquors resulting from cellulose isolation allows for a reduction in water consumption, wastewater volume, and, most importantly, reagent and energy costs, while increasing the resource-saving stability of the process [43]. The expediency of recycling the nitric acid cooking liquor at the stage of chemical pretreatment of oat hulls was successfully proved in [43], where enzymatic hydrolysis of the resulting substrates was found to be efficient.

However, recycling of the cooking liquor has never been applied before in the process of cellulose isolation from miscanthus.

Therefore, the present study aimed to isolate cellulose from giant miscanthus by the nitric acid method with fivefold recycling of the nitric acid solution.

## 2. Materials and Methods

All reagents and materials used in this study were purchased from JSC Vekton (Saint-Petersburg, Russia). Each cellulose sample was obtained in three independent replicates, and the averages of each parameter (mass fraction of components, degree of polymerization, and yield) are presented (see Results and Discussion). The experimental results (cellulose isolation from the feedstock) were statistically analyzed using one-way analysis of variance (ANOVA) with Microsoft Excel 2019. The ANOVA results revealed no significant differences between the groups (*p* > 0.05), with the empirical F value not exceeding the critical value.

### 2.1. Feedstock

The substrate for this study was giant miscanthus stems grown in Penza Oblast (Russia). The feedstock was an average sample from two miscanthus stem batches harvested from plantations in their 7th and 11th years of vegetation. The feedstock was milled using a KR-02 fodder chopper (TekhnoMash Ltd., Miass city, Russia) to a particle size of 3–10 mm. Particles smaller than 3 mm were removed by sieve analysis and discarded. It should be noted that giant miscanthus stems are very strong and difficult to mill.

### 2.2. Compositional Analysis of the Feedstock

Standard analytical methods―so-called “wet” methods―were employed to quantify the components of giant miscanthus. These methods are based on feedstock fractionation and are most commonly used for cellulosic biomass [9].

The cellulose content was determined by the Kürschner method by which a weighed sample of miscanthus is treated with mixed ethanol/nitric acid in a 4:1 ratio [44,45]. The acid-insoluble lignin content was determined using 72% sulfuric acid in accordance with TAPPI standard [46]. Pentosans were quantified by converting them to furfural in boiling 13% HCl solution. The furfural was collected in the distillate, and the optical density was measured using a UNICO UV-2804 spectrophotometer (United Products & Instruments Inc., Dayton, NJ, USA) with xylose calibration at a wavelength of 630 nm using an orcinol–ferric chloride reagent [47]. The ash content was determined as per TAPPI standard [48]. The content of extractives was determined according to TAPPI standard [49] after sequential extraction of a weighed feedstock sample with methylene chloride in a Soxhlet apparatus. The moisture content was measured using an OHAUS MB-25 moisture analyzer (Parsippany, NJ, USA).

### 2.3. Isolation of Cellulose Samples

Cellulose was isolated by the nitric acid method involving sequential treatment of the feedstock with dilute nitric acid (65.0%, CAS No. 7697-37-2, CHIMMEDSNAB, Korolev city, Russia) and sodium hydroxide solutions, with a modification of the main stage—the nitric acid treatment of the feedstock. For the first time, the dilute nitric acid solution was reused fivefold during the treatment of fresh feedstock portions, with the concentration of the spent liquor being increased if necessary.

The feedstock loading for each of the five batches was 200 g. The first stage of the process—the nitric acid treatment of the feedstock—was carried out in a 4 L round-bottom flask fitted with a reflux condenser. The flask was placed into an LTHS 4000 heating mantle (Brněnská Drutěva, Brno, Czech Republic), and the feedstock was held in a 4% HNO_3_ solution for 8 h at 90–95 °C, with a solid-to-liquid ratio of 1:15.

The reaction mixture was then cooled and filtered using a Laboport N811 vacuum pump (KNF, Berlin, Germany). The filtered cooking liquor was collected into a separate container, and its acidity was determined by potentiometric titration [50]. If necessary, the liquor was strengthened with 65% HNO_3_ to a concentration of a 4% HNO_3_ solution and used for subsequent treatment of a fresh batch of miscanthus stems. The acidity of the spent nitric acid solution and the amount of nitric acid required for its strengthening are specified in Table 1.

Thus, the solution was reused five times (treatment nos. I–V, Table 1).

The product obtained after nitric acid treatment was washed on a filter with water until neutral wash waters were reached and then thoroughly squeezed. The product was then subjected to alkaline treatment. The alkaline treatment was carried out with a 4% NaOH solution in a 4 L round-bottom flask equipped with a reflux condenser, in a water bath, for 6 h at 90–95 °C, with a solid-to-liquid ratio of 1:15.

After completion of the process, the resultant cellulose was washed on a filter with water until the washings were neutral. Decationization was performed by holding the product in a 1% HNO_3_ solution for 3 h at 70 °C with a solid-to-liquid ratio of 1:10, followed by final washing on the filter with water until the washings were neutral; the product was then thoroughly squeezed. A portion of the cellulose was dried to determine the composition and degree of polymerization.

### 2.4. Composition and Degree of Polymerization of Cellulose Samples

The contents of residual lignin, pentosans, ash, and moisture were determined by the methods described in Section 2.2.

The α-cellulose content was determined according to the TAPPI standard by treating a cellulose sample with a 17.5% NaOH solution and quantifying the undissolved residue after washing with a 9.5% NaOH solution and water, followed by drying [51]. The degree of polymerization (DP) was determined by measuring the efflux time of a cellulose solution in cadoxene (cadmium oxide in ethylenediamine) using a VPZh-3 viscometer (Ecroshim Ltd., Saint-Petersburg, Russia) with a capillary diameter of 0.92 mm [14,52]. The moisture content was measured by an OHAUS MB-25 moisture analyzer (Parsippany, NJ, USA).

### 2.5. Structural Characterization of Cellulose from Giant Miscanthus Stems by SEM, FTIR Spectroscopy, Coupled TGA/DTA/DTG of Giant Miscanthus Stems and Isolated Cellulose, and X-Ray Diffraction of Cellulose

The surface morphology of ground *Miscanthus* and fibers was examined by scanning electron microscopy (SEM) (Jeol Ltd., Tokyo, Japan) after sputtering Ag with a layer thickness of 1–5 nm. Optical microscopy images of the feedstock and isolated cellulose are also presented.

The molecular structure of *Miscanthus* and cellulose was examined by Fourier-transform infrared (FTIR) spectroscopy using an Infralum FT-801 spectrometer (NPF Lumex Sibir Ltd., Novosibirsk, Russia) single-pass attenuated total reflectance (ATR) in the frequency range of 4000–500 cm^−1^.

The thermal behavior of *Miscanthus* and cellulose was studied by coupled thermogravimetric and differential thermal analysis (TGA/DTA/DTG) using a Shimadzu DTG-60 thermogravimetric analyzer (Shimadzu, Kyoto, Japan) under the following conditions: 0.5 mg sample weight, 10 °C/min heating rate, 350 °C maximum temperature, and nitrogen as an inert atmosphere.

The X-ray diffraction patterns of the studied cellulose obtained on a DRON-3M diffractometer (The Bourevestnik, JSC, Saint-Petersburg, Russia) in reflection and transmission geometries with FeKα radiation, monochromatized by a pyrolytic graphite crystal placed in the primary beams. The X-ray diffraction profiles were scanned over a 2θ range from 3° to 90°, with a step size of 0.1°. The signal counting time per point was 10 s. The full-profile analysis of polycrystal X-ray diffraction patterns (the Rietveld method) established that the cellulose samples correspond in structure to the monoclinic phase of cellulose Iβ. The crystallinity degree (CD) was determined using a modified Ruland method, which is based on separating the peaks corresponding to scattering from the crystalline component of biopolymers and the maximum of diffuse scattering from the amorphous component, followed by fitting the profiles of the calculated and experimental curves [53,54,55,56].

$$\mathrm{CD}=\frac{I_{c}-I_{\mathrm{am}}}{\mathrm{Ic}}*100\%,$$

where *I_c_* is the total integrated scattering intensity from the crystalline and amorphous phases and *I_am_* is the integrated scattering intensity from the amorphous phase.

## 3. Results and Discussion

The compositional profile is a key indicator for assessing the potential of a plant feedstock for its efficient industrial conversion into value-added products, including cellulose [1,6,7,8,9]. The cell wall of giant miscanthus consists mainly of polymers such as cellulose, hemicelluloses, and lignin [6,7,8,9].

There are guidelines for assessing the quality of biomass from mature plants after a growth period of at least 2–3 years [8], and the superiority of miscanthus stems over the leaves for chemical processing due to their higher cellulose content has repeatedly been confirmed [28,57,58]. In the present study, a very high-quality feedstock was used as the substrate for the study: first, giant miscanthus stems, and second, those harvested from a plantation aged 7–11 years. The compositional profile of the giant miscanthus stems is presented in Table 2.

It follows from the data in Table 2 that giant miscanthus stems are characterized by a high Kürschner cellulose content of 52.8 wt%, with a moderate content of non-cellulosics: 26.1 wt% lignin, 19.2 wt% pentosans, 2.06 wt% ash, and 0.51 wt% extractives. The obtained data are in agreement with [15,28,57,59] and surpass those for reported in other studies [60,61,62,63]. For example, the cellulose content of giant miscanthus was found to be 48.4 wt% in Belgium [60], 41.8 wt% in Greece, in 37.8 wt% France, 42.3 wt% in Germany, 49.5 wt% in the United Kingdom [61], 41.6 wt% in the USA [63], and 49.4 wt% in [62].

Due to the high Kürschner cellulose content, the conversion of giant miscanthus stems into cellulose is justified.

Table 3 presents the quality attributes of cellulose samples obtained by the nitric acid method with fivefold recycling of the same cooking liquor at the first stage of the process, as well as the cellulose yield.

It follows from Table 3 that the fivefold reuse of the nitric acid cooking liquor made it possible to obtain cellulose samples of consistently high and mutually comparable quality. The high α-cellulose contents of 92.0–94.0 wt% and DP values of 1750–1850 were achieved. The high degree of polymerization values of cellulose determined by the cadoxen method do not cause doubts, since this method was previously validated through comparison with other methods and was recommended for cellulose samples of various origins, including bacterial nanocellulose [52]. Recently, this method has gained particular popularity and is used for determining the DP of cellulose from algae, terrestrial plants, and bast fibers, over a range from nanocrystalline cellulose (70–110) to bacterial nanocellulose (5000–6200). The content values of non-cellulosics were 0.41–1.06 wt% for lignin, 3.12–3.82 wt% for pentosans, and 0.13–0.35 wt% for ash. This finding justifies the use of the modified nitric acid method with fivefold recycling of the spent liquor at the main stage.

This method ensures the isolation of high-quality cellulose in a 36% yield. At the same time, the content of non-cellulosics were reduced manifold: specifically, lignin content was reduced by 24.6–63.7 times, pentosans were reduced by 4.0–6.2 times, and ash was reduced by 5.9–15.9 times, corresponding to their extraction degree of 98%, 81% and 90% on average, respectively. The average cellulose yield of 36.0% on a feedstock basis corresponds to 68.2% of the native cellulose in the feedstock.

Figure 1 shows photographs of (a) giant miscanthus stems and isolated cellulose and optical microscopy images of (b) giant miscanthus stems and (c) isolated cellulose.

The appearance of the initial feedstock (Figure 1a) represents hard stems 6–7 mm in diameter, with a soft core inside and a rather thick, strong surface, which makes them difficult to mill. The cellulose isolated from giant miscanthus appears as a loose fibrous mass of milky white color. The optical microscopy images (Figure 1b,c) allow the observation of the transformation of the complex-shaped particles of miscanthus stems into delicate clusters of fine cellulose fibers.

Thus, it was discovered that recycling of nitric acid at the main stage of the nitric acid process for cellulose isolation from miscanthus stems enables the isolation of cellulose without impairing its quality attributes.

We previously reported a successful fivefold recycling of a 4.4 wt% nitric acid solution for oat hulls to obtain a nitric acid pretreatment product and found that carrying out more than five treatments leads to a sharp decrease in the cellulose content and to an increase in the contents of lignin and pentosan in the samples from nitric acid pretreatment [43]. In the study [43], only a single-stage nitric acid treatment of the feedstock was performed to obtain a good-quality substrate with high enzymatic digestibility. In contrast to [43], the present study employed a two-stage treatment of giant miscanthus in order to obtain high-quality cellulose for subsequent chemical modification. Thus, the obtained results advance the field of non-wood feedstock processing with dilute nitric acid solution and prove the possibility of producing not an intermediate product, but a target high-viscosity cellulose. It should be noted that giant miscanthus is a significantly more complex feedstock than oat hulls because it has a rigid stem that is difficult to comminute and is more resistant to chemical treatment, as opposed to inherently small and uniform oat hulls.

Furthermore, in our view, the results obtained in this study are priority ones for the cellulose isolation from *Miscanthus*, since recycling of the cooking liquor has not been trialed on this type of feedstock before.

The successful experience in cellulose isolation from *Miscanthus sacchariflorus* by the nitric acid method demonstrated that high-quality cellulose could be obtained with a yield of 23–30% on a feedstock basis [40]. Compared to [40], the cellulose yield from giant miscanthus in the present study is 40% higher and amounts to 36.0%, which quite positively characterizes this method with recycling of the nitric acid cooking liquor. The cellulose yield of 36% is quite high for cellulose with an α-cellulose content above 92%, a DP of at least 1750, and an acid-insoluble lignin content of no more than 1.06%. A higher yield inevitably leads to a decrease in cellulose quality.

A comparison of our data with previously published results from other authors is presented in Table 4. A comprehensive comparison implies the evaluation of the reagent concentration, temperature and time of the feedstock treatment, as well as the cellulose yield based on a feedstock basis, together with the cellulose quality (α-cellulose content, residual lignin, DP). It should be noted that no data on reagent recycling during *Miscanthus* treatment are available in the literature.

The short-term two-stage autoclave treatment of the milled *Miscanthus sinensis* fraction with water first and then with 2.5% sodium hydroxide results in cellulose with a yield of 50.0% and a satisfactory residual lignin content of 4.20%. The study [53] does not provide the cellulose degree of polymerization (DP) and employs the product as an effective substrate for fermentable sugars. The organosolv treatment of giant miscanthus with ethanol at 170 °C [64] affords cellulose with the α-cellulose and lignin contents of 50.01–63.09 wt% and 14.30–16.28 wt%, respectively. The study [64] does not provide the cellulose yield or DP, with the cellulose further used as a substrate for enzymatic hydrolysis. In the study [65], a cellulose yield of 80% was achieved by alkaline hydrodynamic cavitation treatment of giant miscanthus stems, with the α-cellulose and lignin contents being 60.75% and 15.90%, respectively. The other authors [59] used two methods for cellulose isolation from giant miscanthus: soda pulping yielded 78.7–85.7 wt% α-cellulose and 3.4–8.4 wt% residual lignin, while kraft pulping yielded 74.3–82.0 wt% α-cellulose and 3.7–8.7 wt% residual lignin. Unfortunately, the cellulose yield data were not provided, but it can be suggested that these values are much higher than the value of 36.0% achieved in our study. It should be noted that the DP values were not reported in those studies, since the authors did not aim for a low residual lignin content in the obtained cellulose, and DP determination in cellulose with a residual lignin content of 8–9% is impossible due to the insolubility of this cellulose in cadoxene. The quality of the cellulose samples obtained in those studies [59,65] was intended for use in the paper industry.

Previously, we conducted studies on cellulose isolation for the paper industry from giant miscanthus using the same reagents but in the reverse order [66], which made it possible to obtain cellulose with a higher yield of 40% but with a lowered quality, specifically: an α-cellulose content of 92.0–92.6%, a lignin content of 1.0–1.1%, a pentosan content of 10.8–11.2%, and an ash content 0.21–0.30%. If the goal is to obtain high-quality cellulose, i.e., suitable for subsequent chemical modification, priority has to be given to the product quality rather than the yield.

Since all five cellulose samples (Table 3) were very close to each other, the remaining masses of the cellulose samples stored in the wet state at 5 °C were pooled, and the pooled sample was analyzed (a pooled cellulose sample (I–V), Table 3). As expected, the quality attributes of this sample corresponded to the range of values obtained for the individual cellulose samples produced by fivefold recycling of the nitric acid cooking liquor, namely: 93.0% α-cellulose content, 0.74% lignin content, 3.64% pentosan content, and 0.22% ash content (Table 3).

The practical value of the results obtained in this study lies in experimental evidence that high-polymer cellulose with a DP reaching 1850 can be obtained from giant miscanthus, which significantly expands the potential applications of this cellulose and its chemical modification products [17,22,23,24,25,26,27].

Thus, for the first time, high-quality, high-viscosity cellulose was obtained from giant miscanthus stems by the modified nitric acid method with recycling of the nitric acid cooking liquor, with an α-cellulose content of 92.0–94.0% and a DP of 1750–1850.

The use of recycling enabled the cellulose isolation under the following conditions: in the treatment of 1000 g (200 g × 5) of the feedstock, 184.6 g of nitric acid was used to prepare a 4% nitric acid solution at the first stage, and 61 g was used to strengthen the spent liquors. As a result, 360 g of pure cellulose was obtained by using 245.6 g of 65% nitric acid. Without recycling, 923 g of 65% nitric acid would have been required, which is nearly four times more. These data evidence the savings in nitric acid and water during the cellulose isolation from giant miscanthus, which, in general, positively contributes to the reduction in wastewater and environmental impact.

Since all cellulose samples exhibited similar quality parameters, the structural characterization by SEM, FTIR spectroscopy, and TGA/DTA/DTG was performed only for the pooled cellulose sample (I–V) (Table 3) compared to the initial feedstock.

Figure 2 displays SEM images of (a,b,c) giant miscanthus and (d,e,f) the pooled cellulose sample from giant miscanthus (I–V).

The densely formed structure of the single natural “tube” of miscanthus stems suggests the presence of bundles of interwoven fibers inside the tube but they are not currently accessible. Openings, protrusions, and implications of fractures and disruptions of the strong natural structures are visible on the surface of the tube (Figure 2a–c). A similar pattern was observed by Dacres et al. [67], who applied alkaline delignification to miscanthus stems, thereby disrupting the “tubes” and releasing the fibers. By analyzing the undamaged lignocellulosic feedstock, many authors [9,68] have noted a smooth and continuous surface of separate areas of this feedstock compared to the effects of reagents or thermal treatment. In our case, the action of dilute nitric acid and sodium hydroxide solutions on the rigid stems leads to the release of fibers, which become entangled into small fluffy clusters during stirring, squeezing, and drying (Figure 2d). A fivefold magnification in the SEM images (Figure 2e,f) allows one to discern the complex shape of the fibers—resembling round and flat “noodles”—and to note their interweaving with each other.

The cellulose fibers are characterized by heterogeneous shapes distinct in length and width. The fiber width varies from 4 to 18 μm. The cellulose fibers are chiefly intertwined into larger structures—aggregates—but single fibers are also found that are straight, wavy, and twisted multiple times. The bundles of the intertwined cellulose fibers were noted by Dacres et al. [67].

Figure 3 displays the IR spectra of miscanthus (Figure 3a) and the pooled cellulose sample obtained from it (I–V) (Figure 3b).

The IR spectroscopy (Figure 3a) revealed characteristic frequencies indicating the presence of functional groups, including the carbonyl, hydroxyl, carboxyl and methyl groups, corresponding to a lignocellulosic [9,69,70]. The characteristic frequencies of the hydroxyl groups (O–H stretching vibrations) are clearly seen at 3356 cm^−1^, with C–H stretching vibrations observed at 2916 cm^−1^, C=O stretching vibrations observed at 2849 cm^−1^, aromatic skeletal vibrations observed at 1603 cm^−1^, aromatic ring vibrations observed at 1512 cm^−1^, asymmetric angular vibrations of CH_3_ and asymmetric bending vibrations of CH_2_ observed at 1460 cm^−1^, symmetric angular vibrations of CH_3_ observed at 1371 cm^−1^, Ar–O stretching vibrations observed at 1251 cm^−1^, C–O skeletal stretching observed at 1033 cm^−1^, and β-glycosidic bond vibrations of cellulose observed at 896 cm^−1^. These results suggest that the hydroxyl groups link the molecules of cellulose, hemicellulose and lignin together via hydrogen bonding in *Miscanthus*.

The peak at 1731 cm^−1^ corresponds to the acetyl group of uronic acid ester in hemicellulose or to the carboxyester group of the ferulic acid ring and p coumaric acid. In addition, the absorption peak at 1512 cm^−1^ indicates the presence of guaiacylpropane and syringylpropane in lignin of the giant miscanthus stems, suggesting a lignin structure similar to the G- and S-types found in wood of broad-leaved trees [69].

The IR spectroscopy of cellulose (Figure 3b) revealed the presence of the main functional groups characteristic of cellulose [4,21,25,71,72,73]. The highest absorption value is observed at the characteristic frequency near 3337 cm^−1^, corresponding to O–H stretching vibrations in aliphatic structures. The spectrum further shows characteristic bands at 2897, 1644, 1428, 1159, 1054 and 897 cm^−1^, assigned to asymmetric and symmetric stretching of the C–H and CH_2_ groups, O–H bending of adsorbed water, asymmetric CH_2_ bending, C–O–C stretching, skeletal C–O stretching, and the β-glycosidic bond vibration of cellulose, respectively.

The comparison of the IR spectra of the initial feedstock and its cellulose shows that the chemical treatment breaks the bonds between lignin, cellulose, and hemicelluloses in Miscanthus and then disrupts the structure of lignin and hemicelluloses [70]. The IR spectrum of cellulose from giant miscanthus stems shows no peaks of stretching vibrations responsible for impurity components near 1500–1600 cm^−1^ and 1700–1800 cm^−1^, indicating C=C stretching vibrations in the aromatic rings of lignin and C=O stretching vibrations in the ester groups of hemicellulose [4,71], further confirming the high quality of the isolated cellulose.

Figure 4 displays TGA/DTA/DTG curves of giant miscanthus (Figure 4a) and the pooled cellulose sample from giant miscanthus (I–V) (Figure 4b).

Three regions in the TGA curve of the initial feedstock (Figure 4a) show how 2.47% of water is removed from the onset of the experiment through to 100 °C, and the main pyrolysis of the feedstock then occurs between 100 °C and 380 °C. Since the feedstock comprises several polymers and ash components, the TGA curve has a sloping shape, reflecting the gradual decomposition of each polymer (hemicelluloses, cellulose, and lignin), which accounts for a weight loss of 71.74% in total. The shape of the third region in the TGA curve also has a sloping shape, with a weight loss reaching 4.84% between 400 °C and 500 °C. Pyrolysis is typically conducted up to 800 °C, but the main decomposition processes are completed by 500 °C. The comparison of the obtained TGA data for *Miscanthus* stems with those for other lignocellulosic feedstocks shows significant similarities in the TGA curve shape, the presence of three regions, and the correspondence of the maximum decomposition temperature to the range of 350–380 °C [69]. It is well known that hemicelluloses, cellulose, and lignin decompose in different temperature ranges, specifically at 225–350 °C, 325–375 °C, and 250–500 °C, respectively. Therefore, thermally sensitive hemicelluloses decompose first, followed by amorphous cellulose, then crystalline cellulose, with lignin pyrolysis occurring last and over the broadest temperature range. The pyrolysis process ends with slow thermal degradation of residues, leading to the formation of solid residual substances, such as minerals and ash whose contents may reach 15–20%. It should be noted that since lignin is the most thermally stable component of the feedstock due to its complex structure and high stability, the higher the lignin content of the feedstock, the higher the maximum decomposition temperature [74]. The interaction between the polymers directly during the decomposition process also lead to a shift in the maximum decomposition temperature of a specific feedstock compared to the other types. The obtained TGA data are consistent with the component composition of giant miscanthus stems and show similarity to the TGA profile of *Miscanthus floridulus* stems reported in [69].

The DTA/DTG curves of *Miscanthus* stems provide additional information on the decomposition of the feedstock. The first endothermic peak at 49.31 °C is due to the evaporation of bound water on the surface of *Miscanthus* stems, followed by the evaporation of free water and low-boiling-point components. The second endothermic peak at 280.89 °C corresponds to the decomposition of the polymers in the order of their thermal stability. According to Matusiak et al. [75], the general DTA/DTG patterns allow one to distinguish the decomposition of the three polymers with specific temperature ranges: 230–360 °C (with a maximum at ≈296 °C) for hemicelluloses, 285–380 °C (with a maximum at ≈335 °C) for cellulose, and 200–600 °C (with a maximum at ≈381 °C) for lignin, with the main maximum weight loss rate occurring at 334 °C. In the present work, the maximum weight loss rate is 20 degrees higher, i.e., 354.83 °C, which is much closer to the similar temperature of 341.5 °C for giant miscanthus reported in [74].

Similarly to the feedstock, the TGA curve (blue color, Figure 4b) shows that the thermal decomposition of the cellulose sample from giant miscanthus stems occurs in three stages. The first stage demonstrates an initial minor weight loss of 0.98% in the temperature range from the onset of the experiment to 100 °C, which is attributed to the evaporation of adsorbed water that could not be completely removed during drying. This is followed by the main second stage in the temperature range between 100 °C and 400 °C, in which the cellulose sample decomposes with a rapid weight loss of up to 89.66%, accompanied by an endothermic transformation. The onset temperature of intense decomposition of the giant miscanthus cellulose sample was 335.74 °C. This high decomposition temperature indicates that the sample degrades mainly via the cleavage of β-1,4-glycosidic bonds in cellulose. The obtained data are consistent with the observations of other researchers where the cellulose decomposition typically begins at about 275 °C and continues up to 380 °C [71,72,76]. In the third stage, in the temperature range between 400 °C and 500 °C, the cellulose sample continues to decompose with a minor weight loss of up to 3.71%.

The cellulose sample degradation with a major weight loss of 89.66% at up to 400 °C further confirms that complete delignification occurred during the cellulose isolation process because in the presence of a significant amount of lignin, its decomposition would be expected to continue approximately until and after 550 °C [62], leading to a larger weight of residue.

The DTA curve of the cellulose sample from giant miscanthus stems (red color, Figure 4b) shows two endothermic decomposition peaks at 61.17 °C and 364.48 °C: the first corresponds to the removal of water, and the second to the actual decomposition of cellulose. It should be noted that the cellulose sample exhibits a narrow peak, which indicates a more uniform release of bound water due to the high purity of the cellulose sample and its high α-cellulose content. The second peak is very narrow (from 329.75 °C to 385.56 °C), which confirms the thermal stability of cellulose with a low content of non-cellulosics, and is accompanied by absorbed heat of 2.67 kJ/g. The comparison of the obtained results with the published TGA/DTA/DTG data for other cellulose samples obtained from alternative feedstocks [62,63] shows no fundamental differences in the TGA/DTA results; in particular, the maximum DTG peak (cellulose) typically falls within the range of 334–342 °C [74].

Figure 5 presents the X-ray diffraction pattern of the pooled cellulose sample from giant miscanthus (I–V).

The diffraction pattern in Figure 5 shows the characteristic reflections of cellulose I at 14.4–16.2° 2θ associated with (1̅10)/(110), a pronounced peak near 22.3° 2θ associated with the (200) plane, and a small peak at 34.3° 2θ associated with the (004) plane. The high and sharp peak at 22.3° 2θ indicates a high degree of crystallinity due to the treatment of the semicrystalline feedstock with dilute solutions of nitric acid and sodium hydroxide, resulting in the formation of a large amount of stable crystalline cellulose. The treatment of giant miscanthus (the diffractogram of giant miscanthus is not presented) removed the amorphous polymers―hemicellulose and lignin; therefore, the cellulose diffractogram shows the reflections of crystalline cellulose. The study [69] discussed the diffractograms of three parts of *Miscanthus floridulus* and noted that the XRD curve reveals distinct diffraction peaks near 2θ = 16°, 22°, and 35° in the X-ray diffraction patterns of the stem, leaf, and tassel. These peaks correspond to the (110), (200), and (004) crystal planes, respectively, indicating that all three plant parts exhibit the typical virgin cellulose type I structure. The study [69] reported the crystallinity values of native *Miscanthus floridulus*, namely, the crystallinity of the stem, leaf, and tassel yielding values of 48.86%, 46.43%, and 40.70%, respectively (calculated by the Segal method). The same study notes that the crystallinity of cellulose derived from the above-listed parts of miscanthus should be approximately 70%. Although our work discusses cellulose with a crystallinity of 70.4% from the giant miscanthus stems, this crystallinity value aligns with the prediction of the study [69] discussing cellulose with a crystallinity of 70% from the *Miscanthus floridulus* stems. It should be emphasized that the cellulose crystallinity of 70.4% in our work is consistent with the reported value for cellulose isolated from *Miscanthus sinensis* using a 2.0% sodium hydroxide solution (69.6%) [53]. High crystallinity (above 55%) is observed in many cellulose samples isolated from various types of plant raw materials, which is associated with the removal of amorphous hemicelluloses and lignin from the native feedstocks [53,69] rather than with a change in the crystalline structure of cellulose in the native feedstocks. For example, the crystallinity of cellulose isolated from banana rachis is 58% compared with 59% for commercial cotton cellulose [77]. The study [77] underscores that the diffractograms they obtained for both cellulose samples suggest the predominant crystalline form of cellulose—monoclinic Iβ—which is characteristic of the cellulose of terrestrial plants. Myeni et al. [78] discuss the crystallinity of hemp hurds and their cellulose samples, obtained by three different methods, and emphasize that treating the raw material having a crystallinity of 39.26% with a low-concentration 2% sodium hydroxide solution provides cellulose with a crystallinity of 65.80%. It should be noted that those authors attribute the increase in crystallinity of the cellulose samples after pretreatment to the removal of amorphous compounds from the native raw material. Thus, the crystallinity values of cellulose from giant miscanthus stems obtained in our work are comparable with similar studies by many authors, but are lower than the those reported for microcrystalline cellulose from giant reed (79.10%), for cellulose from palm leaves (73.52%), and for cellulose from esparto grass (77.90%) [24], which is entirely logical in view of the high DP values of 1750–1850 for cellulose we isolated.

## 4. Conclusions

In this study, cellulose was isolated for the first time from giant miscanthus stems by nitric acid treatment with recycling (fivefold). All five cellulose samples are characterized by high quality and are very similar to each other. High α-cellulose contents of 92.0–94.0% and DP of 1750–1850 were achieved. The contents of non-cellulosics were 0.41–1.06 wt% for residual lignin, 3.12–3.82 wt% for pentosans, and 0.13–0.35 wt% for ash. The obtained results justify the use of the modified nitric acid method with fivefold recycling of the nitric acid solution at the main stage, which would reduce the reagent and water consumption without compromising the quality attributes of cellulose.

The advantages of this method include a high cellulose yield of 36% on an initial feedstock basis and a high degree of removal of non-cellulosic components: lignin by 98%, pentosans by 81%, and ash by 90%.

The results of characterization of the pooled cellulose sample by SEM, FTIR spectroscopy, and TGA/DTA/DTG are reported. The SEM micrographs revealed a heterogeneous fiber shape, different lengths and widths (ranging from 4 to 18 μm). FTIR spectroscopy confirmed the presence of the main functional groups characteristic of cellulose. The TGA curve shows that the thermal decomposition of the cellulose sample from giant miscanthus stems occurs in three main stages. The DTA curve of the cellulose sample from giant miscanthus stems shows two endothermic decomposition peaks at 61 °C and 364 °C: the first corresponds to the removal of water and the second to the actual decomposition of cellulose. The second peak is accompanied by absorbed heat of 2.67 kJ/g.

Thus, high-quality, high-viscosity cellulose with an α-cellulose content of 92.0–94.0% and a DP of 1750–1850 was obtained for the first time from giant miscanthus stems by a modified nitric acid method with fivefold recycling of the nitric acid solution and can be used for subsequent chemical modification.

## Figures and Tables

**Figure 1:**
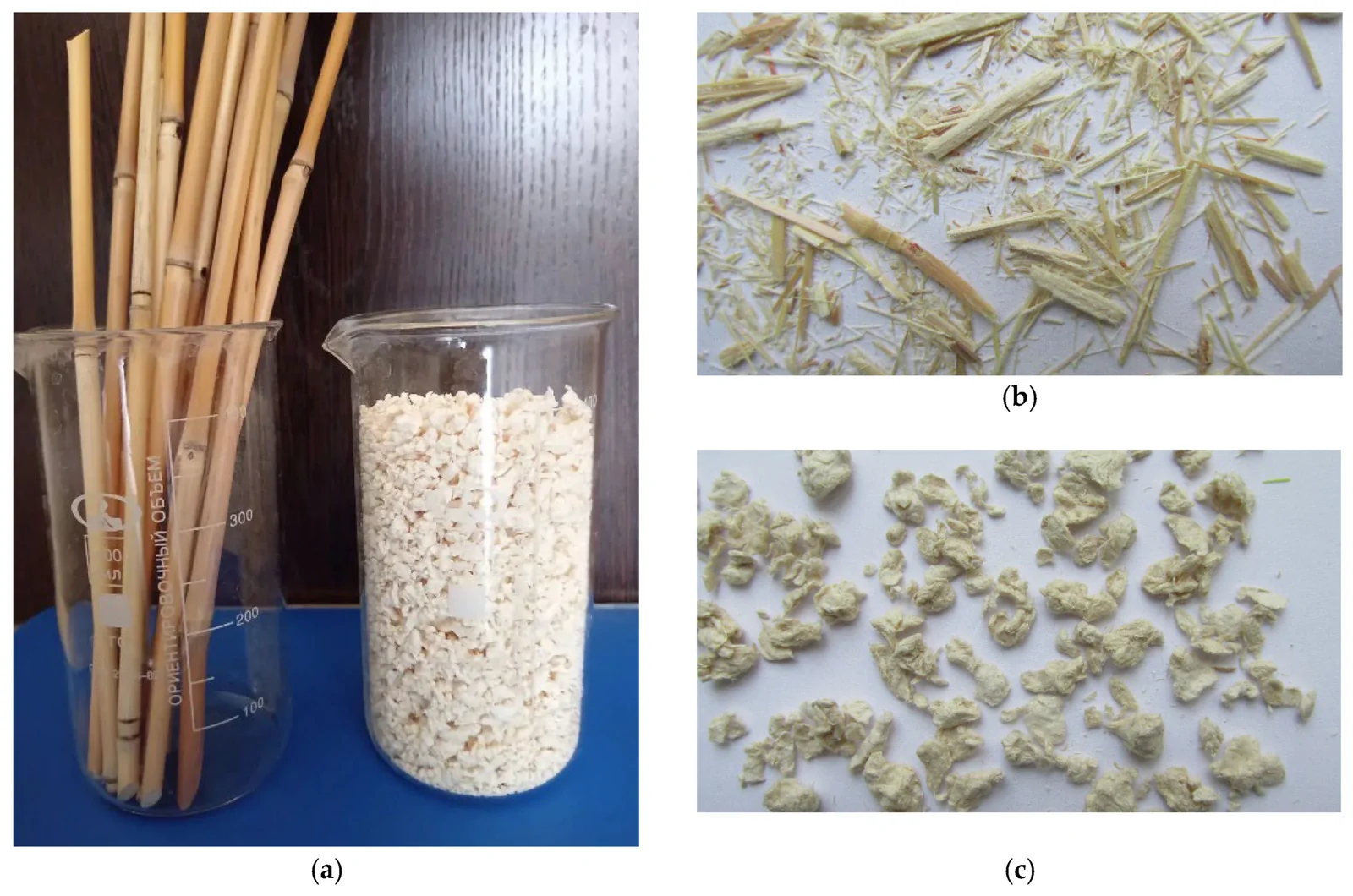
(**a**) Appearance of giant miscanthus stems and isolated cellulose and optical microscopy images of (**b**) giant miscanthus stems and (**c**) isolated cellulose.

**Figure 2:**
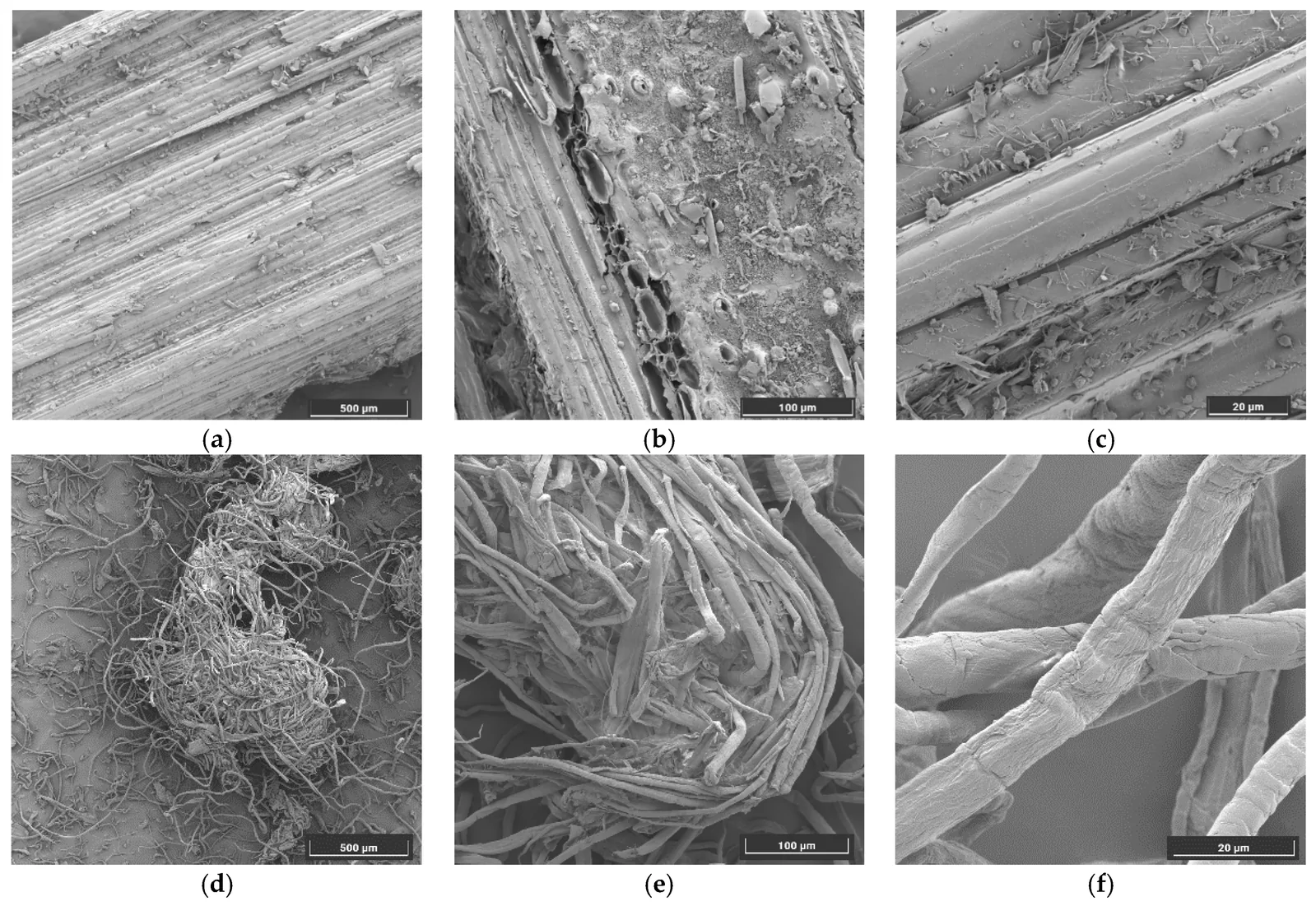
SEM images of (**a**–**c**) giant miscanthus and (**d**–**f**) pooled cellulose sample from giant miscanthus (I–V).

**Figure 3:**
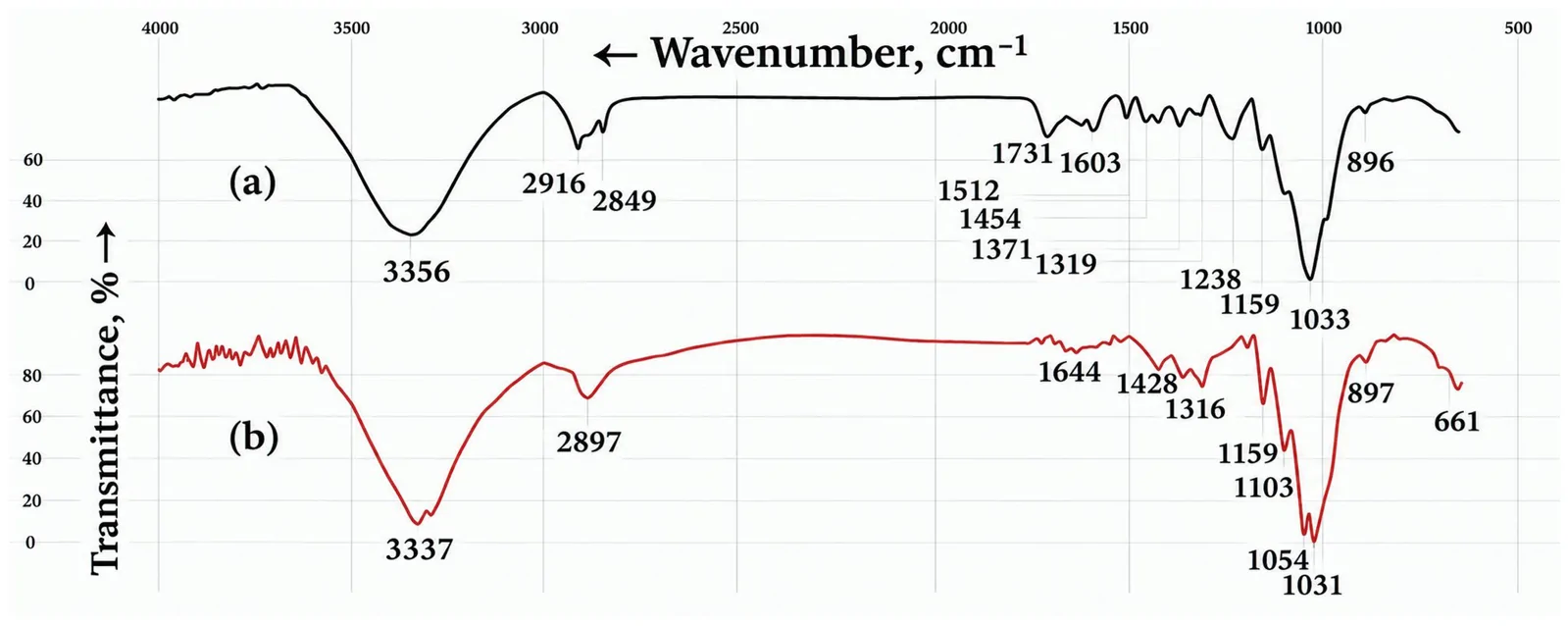
IR spectra of (**a**) giant miscanthus and (**b**) pooled cellulose sample obtained from it (I–V).

**Figure 4:**
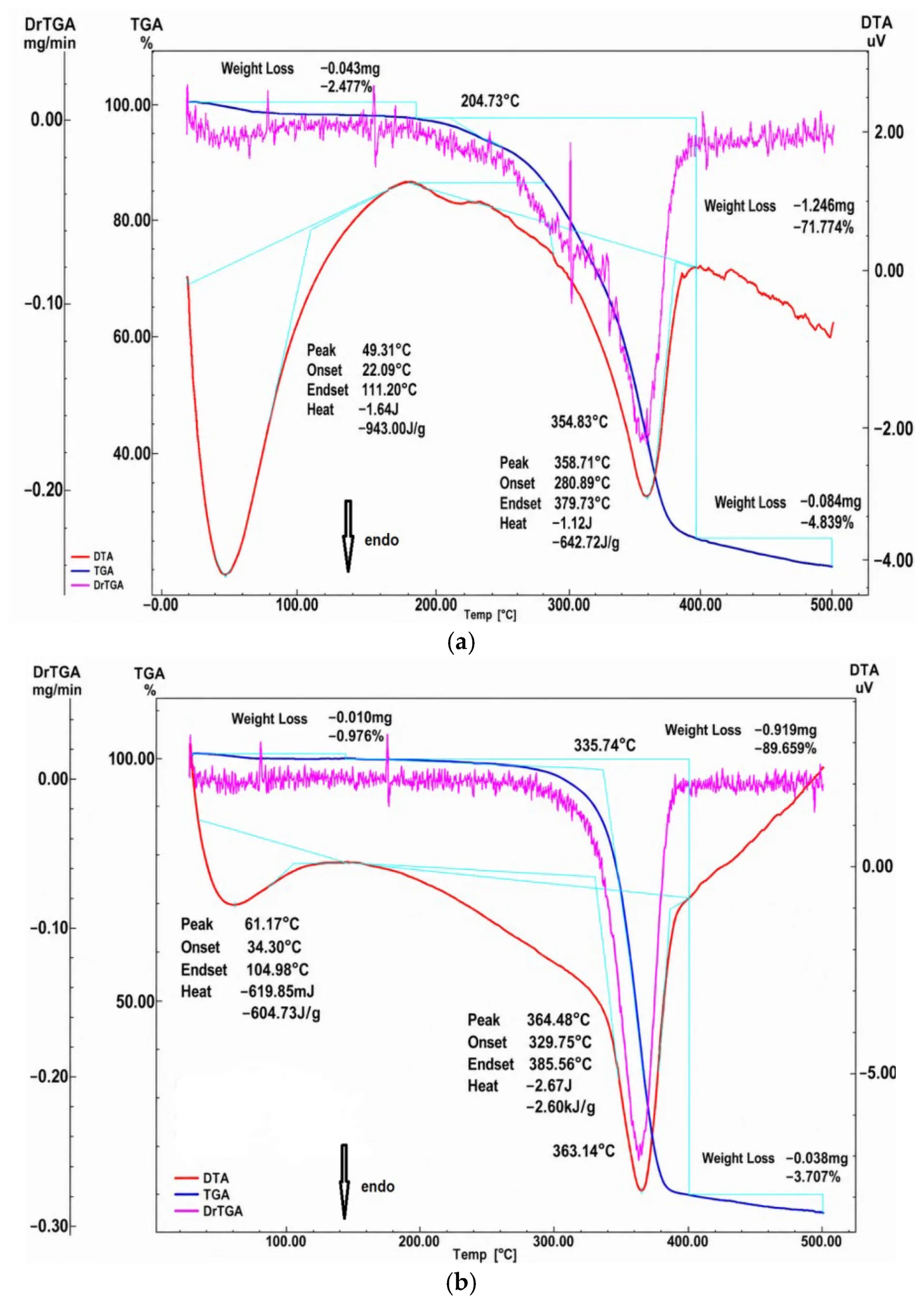
TGA/DTA/DTG curves of (**a**) giant miscanthus and (**b**) the pooled cellulose sample from giant miscanthus (I–V).

**Figure 5:**
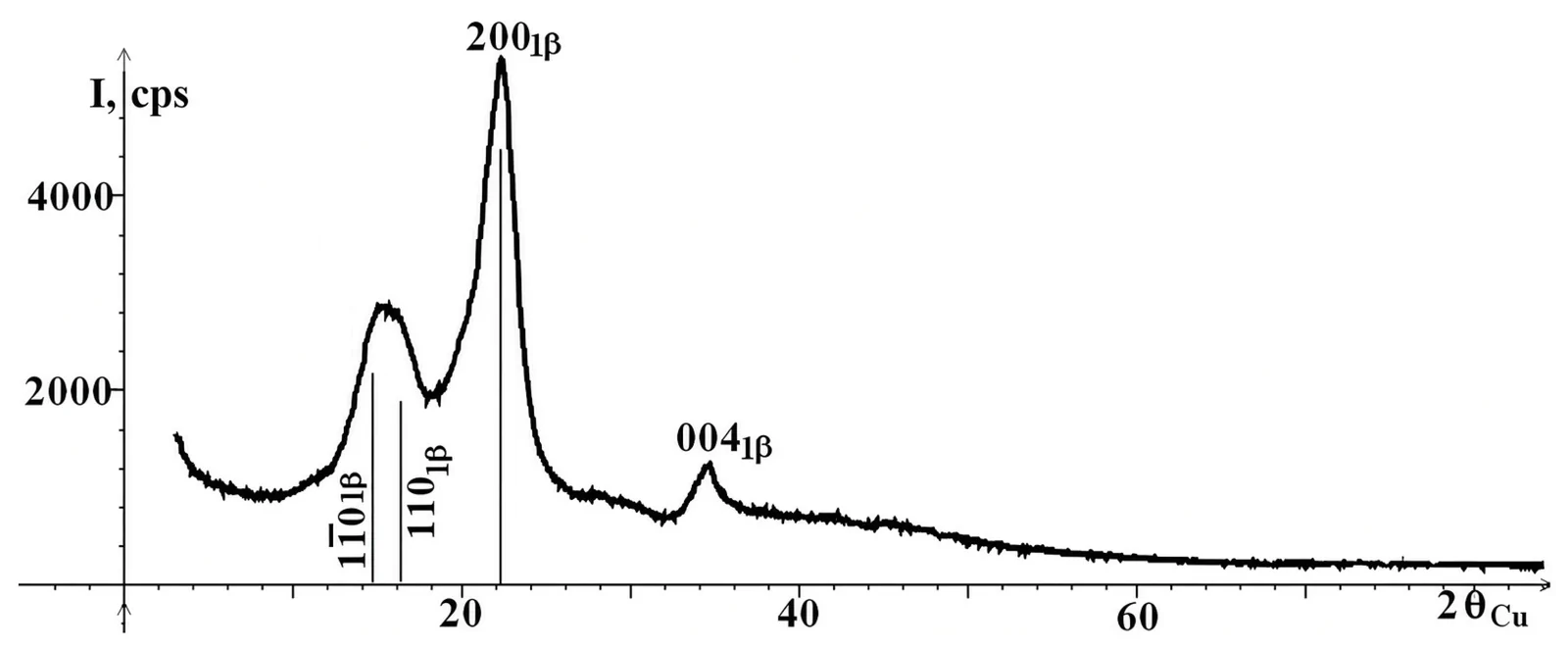
X-ray diffraction pattern of the pooled cellulose sample from giant miscanthus (I–V).

**Table 1:** Acidity of the spent nitric acid solution and the amount of nitric acid required for its strengthening during fivefold reuse.

Nitric Acid Treatment No.	Acidity Before Treatment, %	Acidity After Treatment, %	Amount of Acid Added, g
I	4.00	3.70	15.00
II	4.00	3.70	15.00
III	4.00	3.65	15.50
IV	4.00	3.65	15.50
V	4.00	3.60	-

**Table 2:** Compositional profile of giant miscanthus stems.

Sample	Contents ^1^, wt%				
	Cellulose (Kürschner), %	Lignin, %	Pentosans, %	Ash, %	Extractives, %
Giant miscanthus stems	52.8 ± 0.5	26.1 ± 0.5	19.2 ± 0.5	2.06 ± 0.05	0.51 ± 0.1

^1^ on an oven-dried basis; data are presented as mean ± SD.

**Table 3:** Quality attributes of cellulose samples, obtained by nitric acid treatment of feedstock with fivefold recycling of the first cooking liquor at the main stage of the process, and cellulose yield.

Sample	Contents ^1^, wt%					
	α-Cellulose, %	Lignin, %	Pentosans, %	Ash, %	DP	Yield ^1^, %
Cellulose I	93.0 ± 0.26	0.57 ± 0.06	3.12 ± 0.07	0.30 ± 0.03	1750 ± 50	35.5 ± 0.46
Cellulose II	94.0 ± 0.26	0.47 ± 0.02	3.40 ± 0.09	0.35 ± 0.03	1790 ± 38	36.2 ± 0.26
Cellulose III	92.0 ± 0.26	1.06 ± 0.05	3.76 ± 0.09	0.13 ± 0.01	1850 ± 17	35.7 ± 0.25
Cellulose IV	94.0 ± 0.36	0.94 ± 0.05	3.80 ± 0.02	0.13 ± 0.02	1840 ± 36	36.5 ± 0.17
Cellulose V	92.0 ± 0.15	0.41 ± 0.01	3.82 ± 0.06	0.20 ± 0.04	1800 ± 36	36.1 ± 0.36

^1^ on an oven-dried basis; DP, degree of polymerization; data are presented as mean ± SD.

**Table 4:** Comparative characteristics of cellulose isolation from giant miscanthus stems with recycling versus published data from other studies.

Feedstock	Treatment Conditions	Cellulose Yield, %	α-Cellulose, wt%	Residual Lignin, wt%	DP	Reagent Recycling	Applications	Ref.
*Miscanthus giganteus*	4% HNO_3_ solution for 8 h at 90–95 °C; 4% NaOH for 6 h at 90–95 °C; 1% HNO_3_ solution for 3 h at 70 °C	35.5–36.5	92.0–94.0	0.41–1.06	1750–1850	+	chemical modification of cellulose	this study
*Miscanthus sinensis*	H_2_O 10 min at 105 °C; 2.5% NaOH for 10 min at 105 °C	50.0	70.0	4.20	–	–	fermentable sugars	[53]
*Miscanthus giganteus*	Combined ball milling and organosolv treatment with ethanol at 170 °C for 30–120 min	–	50.01–63.09	14.30–16.28	–	–	increase in enzymatic digestibility	[64]
*Miscanthus giganteus*	13.0–24.0% NaOH at 165 °C for 90 min	–	78.7–85.7	3.4–8.4	–	–	paper industry	[59]
	Soda pulping and kraft pulping, 10.0–20.5% NaOH at 165 °C for 90 min	–	74.3–82.0	3.7–8.7	–	–		
*Miscanthus giganteus*	alkaline hydrodynamic cavitation treatment with 0.3 M KOH for 48 h at room temperature, with periodic milling at 2500 rpm	80.0	60.75	15.90	–	–	paper industry	[65]

## Data Availability

Data are contained within the article.
